# Isolation, Characterization and Genomic Analysis of PBC_MG88 and PBC_MG99 Bacteriophages and Their Antibiofilm Activity Against the *Bacillus cereus* Groups

**DOI:** 10.3390/v18030306

**Published:** 2026-02-28

**Authors:** Maroua Gdoura-Ben Amor, Antoine Culot, Nour El Houda Mathlouthi, Noël Grosset, Clarisse Techer, Sophie Jan, Florence Baron, Hanen Sellami, Michel Gautier, Radhouane Gdoura

**Affiliations:** 1 Research Laboratory of Environmental Toxicology Microbiology and Health (LR17ES06), Faculty of Sciences, Sfax University, BP 1171, Sfax 3000, Tunisia; 2 Rime Bioinformatics SAS, 99120 Palaiseau, France; 3 GenoFlow Agency, Tunis 2036, Tunisia; 4 Science et Technologie du Lait et de l’Oeuf (STLO), l’Institut Agro—l’Institut National de Recherche Pour L’agriculture, L’alimentation et L’environnement (INRAE), 35042 Rennes, France; 5 Mixscience, 35712 Bruz, France; 6 Laboratory of Treatment and Valorization of Water Rejects, Water Research and Technologies Center (CERTE), Borj-Cedria Technopark, University of Carthage, Soliman 8020, Tunisia

**Keywords:** *Bacillus cereus*, bacteriophages, temperate phages, genome analysis, biofilms, biocontrol

## Abstract

*Bacillus cereus* is a major foodborne pathogen responsible for food spoilage and foodborne illness, including strains producing emetic toxins. In this study, two bacteriophages, PBC_MG88 and PBC_MG99, were isolated from wastewater using emetic *B. cereus* strains as hosts and were comprehensively characterized. Both phages formed clear plaques with halos and exhibited siphovirus morphology. Host range analysis against 172 *B. cereus* strains showed that PBC_MG88 and PBC_MG99 infected 50 and 60 strains, respectively. One-step growth experiments revealed efficient lytic activity, with latent periods of 20–25 min and burst sizes of 59–63 PFU per infected cell. More than 90% of phage particles adsorbed to host cells within 15 min. Both phages were stable across a wide temperature range (4–55 °C) and pH values (4–11). Genome sequencing revealed ~37 kb double-stranded DNA genomes lacking antibiotic resistance or virulence genes; however, the presence of lysogeny-related genes suggests a temperate lifestyle. Comparative genomic analyses indicated that both phages represent novel species within the genus *Lwoffvirus*. Biofilm assays demonstrated significant inhibition of *B. cereus* biofilm formation and reduction of pre-established biofilms. Overall, this study expands knowledge of *B. cereus* phage diversity and highlights the importance of genomic characterization in phage-based biocontrol research.

## 1. Introduction

The extensive use of antibiotics for treating infections in humans and animals, as well as their use as growth promoters in livestock, contributes to the emergence and spread of multidrug-resistant (MDR) bacteria, posing a serious threat to public health [1,2]. Some MDR bacteria, with the capacity to form surface-attached biofilms, gain enhanced protection against antibiotics and harsh conditions, increasing their persistence and enabling the transfer of resistance genes [3,4,5,6].

In the food industry, biofilm formation can lead to persistent contamination and equipment damage, resulting in significant sanitary and economic implications [6,7,8]. Common foodborne pathogens can form biofilms, with some bacterial molecules playing dual roles in both biofilm formation and pathogenicity [3]. Notably, the *B. cereus* group acts as a major contributor to toxin-mediated foodborne illness, due to its ability to produce both emetic and enterotoxigenic toxins and its widespread presence in foods [9]. Its abilities to form spores, adhere strongly to surfaces, and develop biofilms resistant to antimicrobials and cleaning agents enable *B. cereus* to persist in diverse environments and food products [10,11].

In commercial dairy plants for example, it can represent over 12% of the biofilm-associated microflora, contributing to its widespread dissemination throughout production systems [12]. Physical and chemical methods used in the food industry to control bacterial contamination face limitations such food quality alterations, chemical residues, bacterial resistance, and high costs [13,14,15]. Thus, researchers are focused on optimizing cleaning and sanitization protocols to effectively disrupt and prevent biofilm formation, thereby improving food safety outcomes [3,6,7].

Bacteriophages have gained attention as effective biocontrol agents against foodborne pathogens and resilient biofilms. Lytic phages offer a direct alternative to conventional antibiotics by selectively lysing target bacteria without promoting resistance [16,17,18,19,20,21,22,23,24,25,26,27], while temperate phages can be genetically modified to enforce obligate lytic activity or used alongside antibiotics that suppress lysogeny, enhancing bacterial clearance, disrupting biofilms, and even functionally re-sensitizing resistant strains by reducing antibiotic minimum inhibitory concentrations [28,29].

Numerous *B. cereus*-infecting phages have been isolated, characterized, and evaluated for their potential as targeted biocontrol agents to mitigate *B. cereus* contamination in food products and food processing plants [12,30,31,32,33,34,35].

Bacteriophages, also known as phages, are viruses that exclusively infect and destroy bacteria. Their high specificity, natural origin, and compatibility with humans, animals, and plants make them biologically safe agents [36]. These features position phages as promising candidates for farm-to-fork applications, particularly in food safety and processing treatments [37,38].

Phages can be applied at different stages of the food production chain to control foodborne pathogens [39,40]. Their small size enables them to penetrate mature biofilms and reach target cells, where phage-encoded enzymes such as depolymerases, tailspike proteins, and lysins actively degrade cell wall polysaccharides and peptidoglycan to facilitate adsorption and DNA injection [41]. They disrupt biofilms through various mechanisms such as relocation via water channels, local replication, and production of depolymerizing enzymes that degrade the extracellular polymeric matrix, allowing deeper penetration and biofilm eradication [42,43,44].

However, the effective application of phages in food systems requires strategies to ensure their stability [45,46]. The latter is highly variable and influenced by factors such as phage type, food composition, environmental conditions, and the physicochemical properties of the food matrix [47].

Extreme pH [48], high temperature [48,49,50], salinity [48,50], UV irradiation [50,51], desiccation [51], and antiphage food compounds, such as milk caseins and organic acids and tannins from fruits and vegetables [52,53], have all been shown to significantly decrease phage viability and activity.

These factors can damage phage structural proteins (capsid, sheath, tail), cause lipid loss, and induce DNA or RNA alterations [54]. Therefore, protecting phage stability is crucial to maintaining phage viability and treatment efficacy [46].

In parallel, biological parameters such as the adsorption constant, adsorption rate, latent period, and burst size also play a pivotal role in determining the success of phage therapy [55].

Additionally, phage genome analysis plays a crucial role in developing safe and effective phage-based therapeutics [56]. Given their known role in horizontal gene transfer, particularly of antimicrobial resistance and virulence genes [57], phages harboring these elements are carefully excluded from food safety and biocontrol applications. In this context, whole-genome sequencing provides key insights into phage properties, ensuring the absence of genes related to toxins, virulence, antibiotic resistance and lysogeny before their use as antibacterial agents [12,58].

A comprehensive safety assessment also requires consideration of the phage replication cycle, as lytic phages are generally more efficient and safer than temperate ones. Lytic bacteriophages, which specifically infect and lyse bacterial hosts, have demonstrated strong potential as biocontrol agents. Their use in food safety, biofilm removal, and the treatment of infections in both human and veterinary medicine is attributed to their high specificity and ability to eliminate pathogenic bacteria without harming beneficial microflora [59,60,61,62]. These virulent phages have a short replication cycle and rapidly multiply within bacterial hosts, disrupting cellular metabolism and causing lysis [16,63].

From a biosafety perspective, lytic phages are preferred over temperate (lysogenic) phages, as they do not integrate into the host genome or alter the bacterial genotype [64,65]. In contrast, temperate phages pose a risk of horizontal gene transfer by inserting their DNA into bacterial chromosomes, potentially spreading antibiotic resistance or virulence factors and contributing to the emergence of harmful strains [64,66]. This concern arises from lysogenic conversion, a process in which prophage integration confers new genetic traits to the bacterial host. These traits can include toxin production, enhanced virulence, or other adaptive advantages that increase bacterial fitness and pathogenicity [67,68]. Therefore, the use of strictly lytic phages and the exclusion of temperate phages in the food industry is essential to ensure safety and efficacy [64]. Although most studies focus on lytic phages, lysogenic (temperate) phages are widespread and represent a valuable natural reservoir that can be engineered into obligate lytic phage with enhanced lethality and host range for biocontrol applications [29]. To ensure the safe use of temperate phages, whole-genome sequencing is essential to screen for phage-encoded toxin genes or bacterial virulence factors that may be transferred through lysogeny [69]. Accordingly, temperate phages have been applied, either in their native form or following genetic modification, to treat various infections [70,71,72]. Temperate phages can be adapted to target intracellular bacteria, which are generally inaccessible to virulent phages [73]. They serve as versatile platforms for genetic modification and delivery systems, for example by integrating the CRISPR/Cas9 system into the phage genome to enhance delivery to target cells, complementing tail fiber proteins to expand host range, and removing virulence genes from the host strain to prevent toxin contamination and the spread of virulence factors [73].

In this study, two bacteriophages, PBC_MG88 and PBC_MG99, were isolated and characterized with the aim of enhancing the understanding of *B. cereus*-specific phages and evaluating their potential as effective biocontrol agents, particularly for incorporation into phage cocktails designed to prevent and control *B. cereus* contamination in food systems.

## 2. Materials and Methods

### 2.1. Bacterial Strains and Culture Condition

A collection of 174 *B. cereus* group strains, previously isolated from a total of 687 Tunisian food samples [74], was used in this study. Two strains (strains 478 and 3990) harboring emetic toxin-producing genes were used as host strains for phage isolation, while the remaining strains (*n* = 172) were used for host range assays.

*B. cereus* strains were cultured in Brain Heart Infusion (BHI) medium (Fisher Bioblock, Illkirch, France) supplemented with yeast extract (YE) (Fisher Bioblock). All BHI-YE plates or broth cultures were incubated aerobically at 30 °C for 24 h.

#### 2.1.1. Sampling

Bacteriophages were isolated from sewage water collected at a wastewater treatment plant in Rennes, France. Sampling was performed at two points: raw influent entering the plant prior to any processing and effluent collected after tertiary treatment. The collected material was pooled, homogenized, allowed to settle, pre-filtered through gauze, and subsequently passed through a 0.22 µm sterile membrane filter (Sarstedt, Nümbrecht, Germany) to remove debris and bacterial cells.

#### 2.1.2. Phage Isolation and Purification

A 100 µL aliquot of the filtrate was added to 5 mL of each host bacterial culture in early log phase and incubated under agitation at 30 °C for 24 h to enrich for phages.

Each enriched culture was then centrifuged at 10,000× *g* for 20 min at 4 °C and the supernatant was filtered using 0.22 µm filters (Sarstedt, Nümbrecht, Germany). Bacteriophages in the supernatant were tested for plaque formation using the standard double-agar layer method [75,76]. Lysis zones indicated phage presence. A well- separated single plaque was picked, transferred into 3 mL of BHI-YE broth, and left overnight at room temperature to allow phage diffusion from the agar. The suspension was then filtered through a 0.22 μm filter. The double agar plate method was used for further purification, which was repeated several times until a stable and uniform plaque morphology was obtained. Phage-free cultures (containing only bacterial host) and host-free cultures (containing only phage) were used as controls. The two bacteriophages infecting host strains 3990 and 478 were named PBC_MG88 and PBC_MG99, respectively.

#### 2.1.3. High-Titer Phage Stock Preparation

The phage concentration was carried out using a modified method from previous studies [33,76]. Purified phage filtrates were serially diluted tenfold in TS buffer (8.5 g of NaCl and 1 g tryptone per liter) to obtain a concentration that produced confluent lysis of the host using the soft agar overlay method. The dilution of each phage stock that yielded the most confluent lysis was selected and used to inoculate 20 plates via the overlay technique. After overnight incubation, 5 mL of BHI-YE broth was added to each plate. The resulting suspension was collected, incubated at 4 °C for 3–4 h to allow phage diffusion, then centrifuged at 12,000× *g* for 20 min. The supernatant was filtered (0.22 μm) and ultracentrifuged at 150,000× *g* for 2 h at 20 °C. Pellets were resuspended in 300 µL of TE buffer (10 mM Tris-HCl, 1 mM EDTA, pH 8) and stored at 4 °C. Each Phage titer was determined using the soft agar overlay method with the appropriate host strain. Plaques were counted and results were expressed as plaque-forming units per milliliter (PFU/mL).

### 2.2. Host Spectrum Evaluation

The host range of the two phages was determined using a spotting assay [33]. Briefly, 5 mL of BHI-YE soft agar (0.8% agar) maintained at 45 °C was inoculated with 100 µL of each *B. cereus* strain culture (10^8^ CFU/mL) and overlaid onto pre-solidified BHI-YE agar plates (1.5% agar). Once the overlay solidified, 10 µL of each phage suspension (10^8^ PFU/mL) was spotted onto the surface and allowed to air-dry for 30 min. Plates were then incubated overnight at 30 °C. If lysis spots could be formed, the strain was considered as potential host of the phage. Each test was performed in triplicate. As a negative control, each strain was also spotted with sterile phage buffer.

### 2.3. Optimal Multiplicity of Infection Determination (MOI)

The multiplicity of infection (MOI) refers to the ratio of phage particles to bacterial host cells used in the infection assay. The MOI was assessed through a standardized assay with slight methodological modifications [30].

Each *B. cereus* host strain culture in exponential phase was diluted to 10^7^ CFU/mL. One hundred µL of the diluted host bacterial culture were mixed with 100 µL of the corresponding phage at titers of 10^8^, 10^7^, 10^6^, 10^5,^ and 10^4^ PFU/mL, resulting in MOIs of 10, 1, 0.1, 0.01, and 0.001 respectively. A negative control was treated with sterile phage buffer instead of the phage suspension. After 6 h of incubation at 30 °C, the cultures were centrifuged and filtered, and phage titers were determined using the double-layer agar plate method. The MOI yielding the highest phage titer was the optimal MOI.

### 2.4. One-Step Growth Curve Analysis

To characterize the replication dynamics and infectivity of each phage toward its bacterial host strain, a one-step growth curve assay was performed as described by Chen et al. (2024) [30], with some modifications. Briefly, 1 mL of the host bacterial suspension (10^8^ CFU/mL) was centrifuged (10,000× *g*, 10 min, 4 °C) and resuspended in 1 mL Tryptic Soy Broth (TSB), containing tryptone (17.0 g/L), soya peptone (3.0 g/L), NaCl (5.0 g/L), dextrose (2.5 g/L), and K_2_HPO_4_ (2.5 g/L). The phage suspension (10^7^ PFU/mL) and host bacterial culture were preheated at 37 °C for 5 min, then mixed to obtain an MOI of 0.1. At this MOI, approximately 9.5% of bacterial cells are expected to be infected, according to the Poisson distribution [77].

After a 10 min adsorption period, the mixture was centrifuged again to remove unabsorbed phages, and the pellet was resuspended in 1 mL TSB with 1 mM CaCl_2_ to prevent subsequent phage adsorption [66].

Then, 50 µL of this suspension was 1000-fold diluted into 50 mL of fresh TSB (containing 1 mM CaCl_2_) and incubated under agitation at 37 °C. Samples (500 µL) were collected every 5 min within a 50 min period, filtered (0.22 µm), and phage titers were determined using the double-layer agar plate method.

The experiment was performed in triplicate, and a one-step growth curve was generated from the average values. The latent and eclipse period of each phage was directly read and the burst size was calculated as follows:

$$Burst\;size\;=\;\frac{Final\;phage\;amount}{initial\;number\;of\;infected\;bacterial\;cells\;throughout\;the\;latency\;period}$$

### 2.5. Phage Adsorption Assay

Phage adsorption experiments were conducted using an established method with slight modifications [78].

Both phage and bacterial cell suspensions were pre-warmed at 37 °C for 5 min. Subsequently, each phage suspension was mixed with the corresponding host bacterial suspensions in the exponential growth phase (10^8^ CFU/mL) at a multiplicity of infection (MOI) of 0.1 and then incubated at 30 °C for adsorption analysis.

Aliquots (100 uL) were taken every 3 min from 0 to 15 min and filtrated through a 0.22 μm syringe filter. The filtrate containing unadsorbed free phage particles were diluted and enumerated by the by the double agar overlay plaque technique.

Bacteria-free (phage only) and phage-free (bacteria only) setups served as negative controls. The experiments were performed independently in triplicate. Adsorption was expressed as percent decrease of phage titer in the supernatant compared to the initial input dose of phages.

### 2.6. Phage Morphology Observation

Phage morphology was examined by transmission electron microscopy (TEM) following a negative staining protocol used in previous study [31]. Phage suspensions, concentrated by cesium chloride gradient centrifugation and adjusted to a titer >10^10^ PFU/mL, were used to ensure adequate particle visualization. Thirty microliters of phage stock were applied to 200-mesh copper grids coated with carbon-formvar films (Electron Microscopy Sciences, Hatfield, PA, USA) and incubated for 1 min. The grids were subsequently stained with 2% aqueous uranyl acetate for 1 min, excess liquid was removed with filter paper, and the samples were air-dried. Morphological features of the bacteriophages were then visualized using a JOEL JEM-1400 transmission electron microscope (JEOL, Peabody, MA, USA).

### 2.7. Thermal Stability and pH Tolerance Tests of Phages

To determine the thermal stability of PBC_MG88 and PBC_MG99, each phage was suspended in TSB to a final titer of 8 log_10_ PFU/mL (pH 7.0). Aliquots (1 mL) of phage lysate in sterile screw-capped tubes were incubated at 4 °C and in a series of constant temperature water baths at 25, 37, 45, 55, 65, and 75 °C for 1 h. Following incubation, the contents of the tubes were serially diluted and appropriate dilutions were plated to determine the phage titer using the double-layer agar plate method.

For the pH stability test, the pH of TSB was adjusted to values ranging from 3 to 12 using 1 M HCl or 1 M NaOH solution. Phage lysates were suspended to a final titer of 8 log_10_ PFU/mL in the adjusted TSB solutions (pH 3, 4, 5, 6, 7, 8, 9, 10, 11, and 12) and then incubated at 30 °C for 1 h. The residual phage titers were determined by the double-layer agar overlay technique.

All experiments were performed in three biological replicates. Phage stability was calculated using the following formula:

$$Percent\;Stability\left(\%\right)=\frac{N}{N_{0}}\times 100$$

where *N* is the number of viable phages after 1 h of incubation and *N*_0_ is the initial number of phages.

### 2.8. Phage DNA Extraction, Genome Sequencing, and Bioinformatic Analysis

Phage genomic DNA was extracted from high-titer suspensions using the phenol/chloroform method, as described in a previous study [31]. Briefly, samples were treated sequentially with DNase, proteinase K, and SDS, followed by phenol/chloroform/isoamyl alcohol extraction and ethanol precipitation. The resulting DNA was resuspended in Milli-Q water, and its quality was evaluated by NanoDrop ND-1000 spectrophotometry (Nanodrop Technologies, Wilmington, NC, USA) and confirmed by 1% agarose gel electrophoresis.

The extracted DNA was sequenced by GenoScreen (Lille, France) on a MiSeq platform using a paired-end strategy according to the Nextera XT library kit in a 2 × 250-bp format (Illumina Inc., San Diego, CA, USA).

The sequencing reads were subsequently cleaned using Cutadapt v1.18 [79] and Prinseq v.0.20.4 [80]. Assembly was carried on using SPAdes v3.11.1 [81]. Contigs with high coverage (over ×1000), matching with known phages from the NCBI NR database upon blastn query [82], and which size matched with our wet lab measurements using DNA enzymatic digestion were kept. Other contigs were considered as residual DNA contamination from the host strains.

Functional annotation was performed using Multiphate 1.0 pipeline [83]. The dependencies of Multiphate that were called are Phanotate 1.3.1 [84], BLAST 2.9.0+ [82], HMMER3 [85], tRNAscan-SE 2.0 [86], Biopython 1.7 [87], and EMBOSS [88]. Multiphate annotation was achieved using the Nun Redundant database (NR), viral protein database, and viral genomes database from NCBI, PhanToMe genome database [89], Swissprot database [90], and pVOG database [91]. The annotation pipeline was set up with 70% blastp identity threshold, 60% blastn identity threshold, and 0.01 e-value threshold. Protein sequences that could not be annotated by Multiphate were submitted to HHpred for protein remote homology detection and 3D structure prediction [92], using 90% probability threshold and 10^−6^ e-value threshold. The databases used for HHpred were PDB mmCIF70 4 Feb, PDB mmCIF70 4 Feb, SCOPe 70 2.07, and ECOD F70 20190225. Insertion sequences and transposons were searched using ISfinder [93]. Genome maps were generated using CGview online server v1.0 [94]. A blast alignment of the two phages was also performed in order to compare their DNA sequence.

In addition, VIRIDIC was used to calculate intergenomic distances and sequence similarity between the isolated phages and the six most closely related NCBI phages, generating a heatmap that provided a clear visualization of phage relatedness [95].

### 2.9. Anti-Biofilm Assays

Before performing the anti-biofilm assays, preliminary verification experiments were conducted to confirm the biofilm-forming ability of *B. cereus* host strains 478 and 3990 following a previous protocol [96,97]. Briefly, overnight bacterial cultures in BHI were standardized to 10^8^ CFU/mL. A 200 μL suspension of each strain was added in triplicate to 96-well polystyrene microplates, with broth-only wells as negative controls. Plates were incubated at 30 °C for 24 and 48 h. After incubation, wells were washed three times with 300 μL PBS (pH 7.3), heat-fixed at 60 °C for 1 h, and stained with 150 μL of 2% *w*/*v* crystal violet for 15 min. Excess dye was removed by rinsing three times with distilled water and air-dried for 15 min. Biofilms were solubilized with 150 μL of 95% ethanol for 30 min, and absorbance was measured at 595 nm. Mean OD values (ODs) were calculated for each strain, and the cut-off OD (ODc) was defined as three standard deviations above the mean OD of negative controls. Strains were classified as strong (ODs > 4 × ODc), moderate (2 × ODc < ODs ≤ 4 × ODc), weak (ODc < ODs ≤ 2 × ODc), or non-biofilm producers (ODs ≤ ODc) [97]. Three independent experiments were performed.

The inhibitory and disruptive activities of the two phages against biofilms formed by their respective *B. cereus* strains were assessed using plastic 96-well flat bottom plates. Biofilm quantification was performed through a modified colorimetric assay [98].

The overnight cultured host bacteria, grown to exponential phase (10^8^ CFU/mL), were used in subsequent experiments.

Phage suspensions were prepared at 10^7^ PFU/mL, which corresponds to the optimal MOI of 0.1.

To assess the effect of phages on inhibiting biofilm formation in 96-well plates, 100 μL of the host bacteria and 100 μL of phages suspension were added to the wells. The control wells contained 100 μL of bacterial suspension and 100 μL of TE buffer.

Following biofilm formation at 30 °C for 24 h, the wells were emptied by gentle tapping and washed five times with 200 μL sterile phosphate buffered saline (PBS, pH 7.2) to remove planktonic cells and debris.

Wells were then air-dried and stained with 150 μL of crystal violet solution (20% ethanol, *v*/*v*) for 15 min at room temperature. Excess stain was discarded, and wells were rinsed three times with sterile water. To solubilize the bound dye, 200 μL of 33% (*v*/*v*) glacial acetic acid was added to each well and incubated for 1 h at room temperature.

The percentage of inhibition of bacterial adherence and biofilm formation by each phage was calculated using the following formula:

[(OD (growth control)−OD (sample))/(OD (growth control))]×100

For biofilm removal assays, 200 μL of host bacteria was added to each well and incubated at 30 °C for 48 h to allow mature biofilm formation. Non-adherent cells and media were removed, followed by five washes with PBS. Phage suspensions at varying titers (200 μL) were then added, while the controls received 200 μL of TE buffer. After incubation at 30 °C for 6 h, the remaining biofilms were stained with crystal violet as described previously and quantified by measuring absorbance at 590 nm. The percentage of biofilm eradication was calculated according to the formula described above. All assays were conducted in triplicate.

### 2.10. Statistical Analysis

All assays were performed in triplicate, and the results were calculated as the mean ± standard deviation. The statistical significance of the differences between measurements was evaluated using the Student’s t-test, implemented in Microsoft Excel. A *p* value of less than 0.05 (*p* < 0.05) was considered statistically significant.

## 3. Results

### 3.1. Morphological, Biological, and Lytic Characteristics of Isolated Bacteriophages

**Figure 1:**
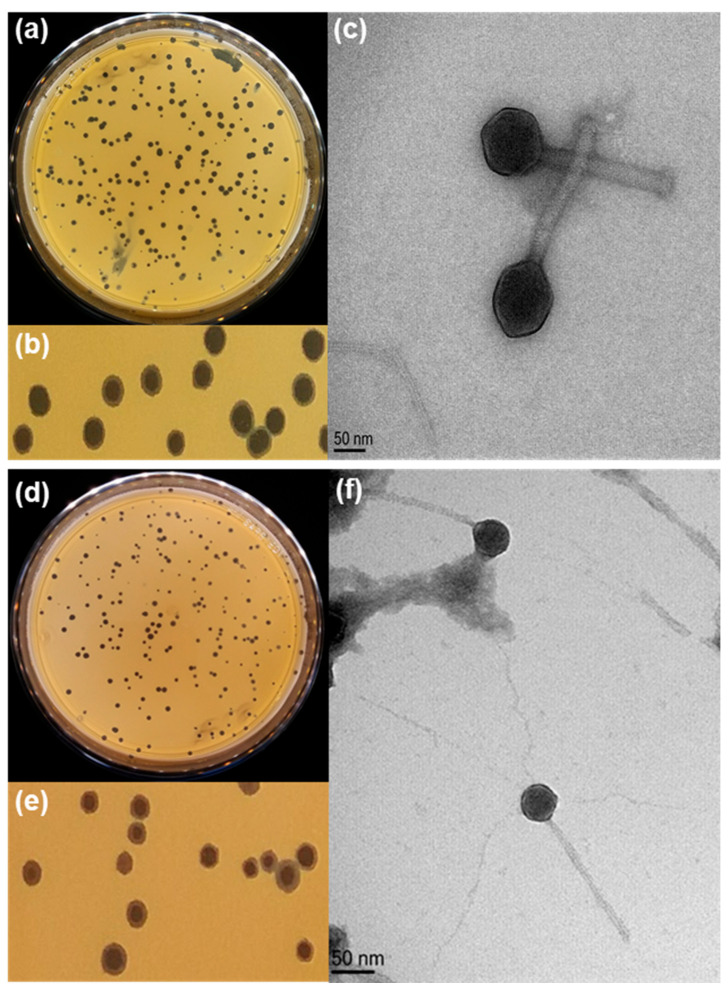
Plaque formation and morphological characteristics of phages PBC_MG88 and PBC_MG99 on *B. cereus* host strains 3990 and 478, respectively. (**a**,**d**) Plaques of phage PBC_MG99 and PBC_MG88, respectively. (**b**,**e**) Enlarged views of plaques formed by phages PBC_MG99 and PBC_MG88, respectively. (**c**,**f**) Transmission electron microscopy images showing the morphology of phages PBC_MG99 and PBC_MG88, respectively. Virions were negatively stained with uranyl acetate and visualized at a magnification of 250,000×. The scale bar indicates 50 nm.

Two phages, named PBC_MG88 and PBC_MG99, were isolated from wastewater using two strains harboring emetic toxin-producing genes as host. Both phages exhibited clear plaque formation on soft agar, with surrounding halos (Figure 1a,b,d,e).

Based on their morphological features observed under transmission electron microscopy (TEM) (Figure 1c,f), both phages exhibited a typical icosahedral head and a long, flexible, non-contractile tail. The head diameters of PBC_MG88 and PBC_MG99 were 85.3 ± 25.7 nm and 74.3 ± 30.7 nm, respectively, while the total virion lengths were 353.4 ± 57.9 nm and 332.0 ± 88.9 nm. In the former morphology-based classification system, such phages were assigned to the family *Siphoviridae* within the order *Caudovirales* according to tail structure [99]. However, these morphology-based taxa have been abolished and replaced by the class *Caudoviricetes*, which belongs to the realm *Duplodnaviria* [100]. According to the latest nomenclature rules of the International Committee on Taxonomy of Viruses (ICTV 2025; https://ictv.global/taxonomy; accessed on 14 August 2025), this class now encompasses all tailed double-stranded DNA (dsDNA) bacteriophages, characterized by an icosahedral head containing the genome and an attached tail structure.

To determine the host range of the phages, 172 *B. cereus* strains isolated from various food sources in Tunisia [74] were tested using the spot assay. Both phages were able to infect a subset of the tested strains (Table S1).

Specifically, PBC_MG88 lysed 50 out of 172 tested strains (29.1%), of which 35 strains (20.3%) produced clear plaques and 15 strains (8.7%) produced opaque plaques. PBC_MG99 lysed 60 out of 172 strains (34.9%), with 48 strains (27.9%) forming clear plaques and 12 strains (7.0%) forming opaque plaques.

**Figure 2:**
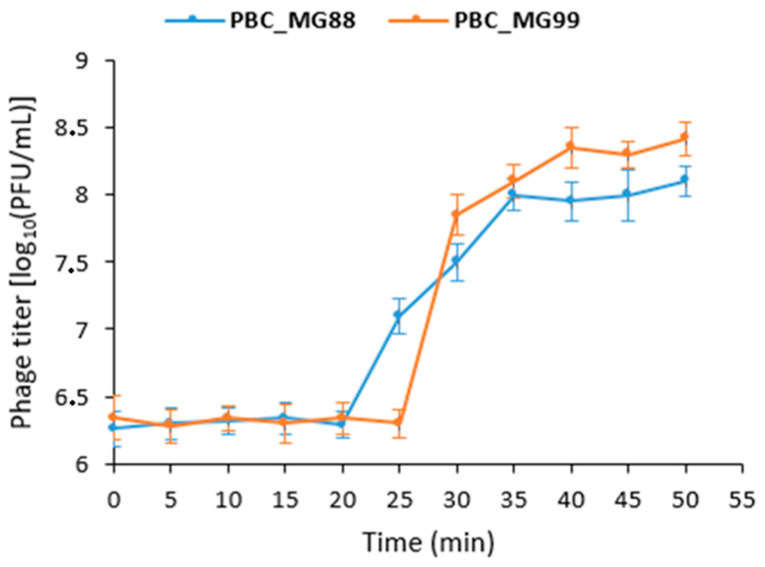
One-step growth curves of PBC_MG88 and PBC_MG99. Phages were grown in exponential-phase cultures of their respective *Bacillus cereus* host strains 3990 and 478. Plaque-forming units (PFUs) per infected cell are shown at different post infection time points. Samples were taken at intervals (every 5 min up to 50 min). The experiment was repeated three times. Data are presented as the mean ± standard deviations from three replicate experiments.

Phage replication dynamics were examined using a one-step growth curve (Figure 2). PBC_MG88 had a latent period of 20 min and an eclipse period of 15 min (20–35 min), followed by a plateau phase, with an average burst size of 59 PFU per infected cell. PBC_MG99 exhibited a latent period of 25 min and an eclipse period of 15 min (25–40 min), entering a stable phase at 40 min, with a burst size of 63 PFU per infected cell.

**Figure 3:**
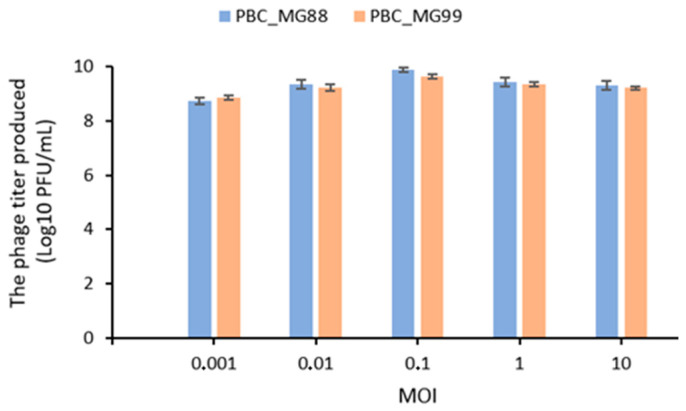
Determination of optimal multiplicity of infection (MOI) of PBC_MG88 and PBC_MG99 phages infecting *B. cereus* host strains 3990 and 478, respectively. Five MOI values (0. 001, 0.01, 0.1, 1, and 10 PFU/CFU) were tested, and phage titers were quantified using the double-layer agar plate method after 6 h of incubation at 30 °C. Results represent the mean ± SD of three independent experiments performed in triplicate.

As shown in Figure 3, phage titers produced by PBC_MG88 and PBC_MG99 varied across MOIs ranging from 0.01 to 10, with an optimal MOI of 0.1 observed for both phages.

**Figure 4:**
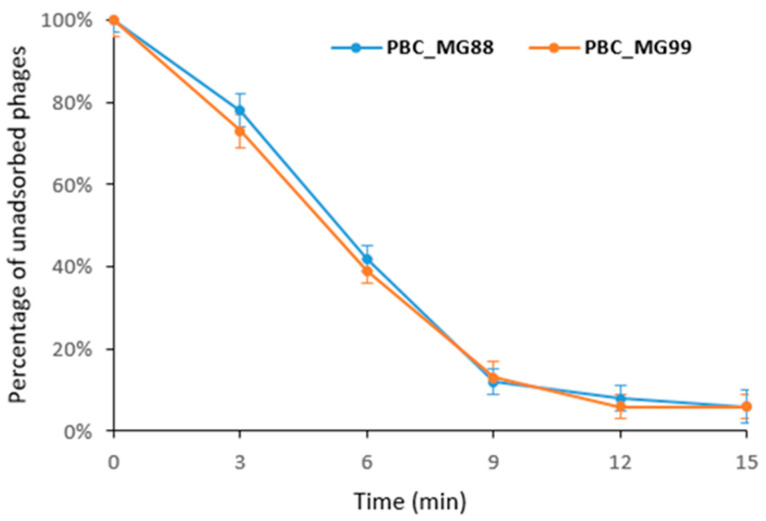
Adsorption rates of phages PBC_MG88 and PBC_MG99 on their respective *B. cereus* host strains 3990 and 478. Each phage was mixed with an excess of its corresponding host strain, and the non-adsorbed infectious phages were quantified by serial dilution. Results are presented as percentages of non-adsorbed phages relative to the initial phage titer and are shown as mean ± SD from three independent experiments performed in triplicate.

The adsorption experiments for both phages revealed that more than 90% of the phage particles attached to the host bacterial cells within 15 min (Figure 4).

**Figure 5:**
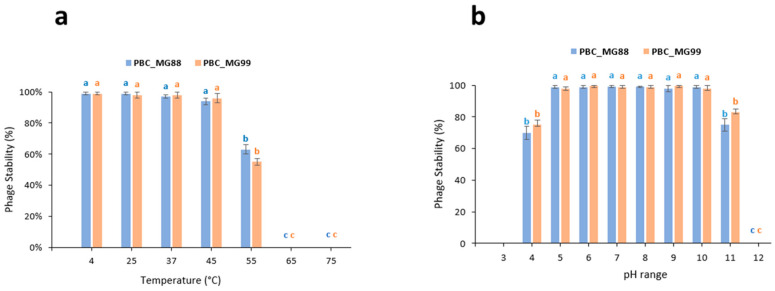
Physicochemical stability of phages PBC_MG88 and PBC_MG99 on *B. cereus* host strains 3990 and 478, respectively. (**a**) temperature stability; (**b**) pH stability. Data are presented as the mean ± standard deviation of three independent determinations. Different letters indicate statistically significant differences among treatments (*p* < 0.05). Blue letters denote comparisons among conditions for PBC_MG88, while orange letters denote comparisons among conditions for PBC_MG99.

Phages PBC_MG88 and PBC_MG99 showed high stability across a wide range of environmental conditions. Both phages maintained their infectivity between 4 and 45 °C, whereas a marked reduction in activity was observed at 55 °C. Complete loss of infectivity occurred after 1 h of incubation at 65 °C and 75 °C (Figure 5a). Moreover, both phages remained stable over a broad pH range, from pH 4 to pH 11 (Figure 5b).

### 3.2. Phage Sequencing and Genome Analysis

The genome sizes of PBC_MG88 and PBC_MG99 are 37 026 bp and 37 328 bp for Ø1BC478, respectively. Table 1 sums up the number of coding sequences (CDS) found during the annotation step of the phages and the mean number of reads containing a given nucleotide (coverage).

**Figure 6:**
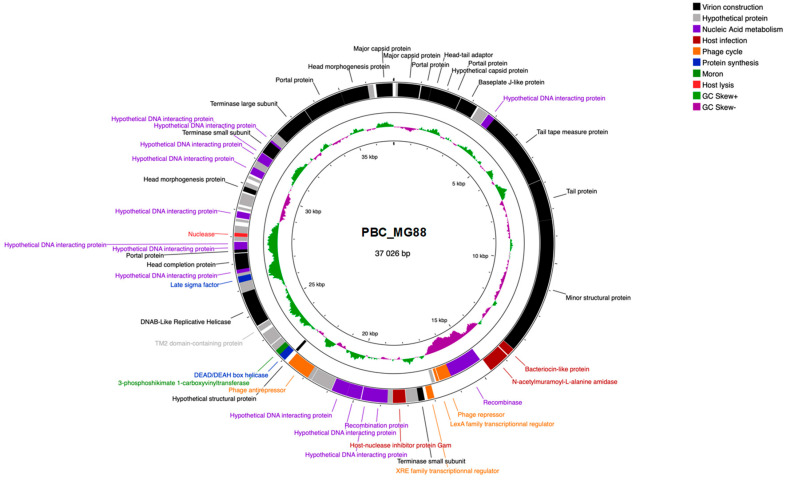
Genome map of phage PBC_MG88. CDS placed on inner and outer rings are read in opposite directions. CDS for which no function could be attributed are classified as “Hypothetical proteins”.

**Figure 7:**
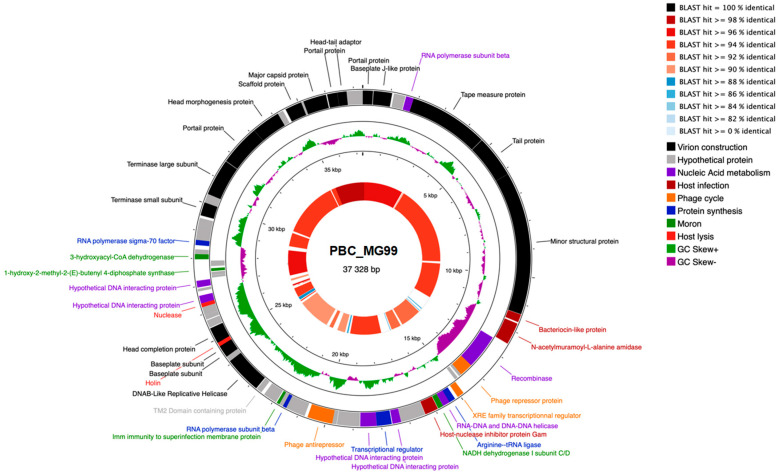
Genome map of phage PBC_MG99. CDS placed on inner and outer rings are read in opposite directions. CDS for which no function could be attributed are classified as “Hypothetical proteins”. The inner ring is the local identity score calculated blastn comparison with phage PBC_MG88 calculated by blastn comparison.

The genome maps of PBC_MG88 and PBC_MG99 are presented in Figure 6 and Figure 7, respectively.

Interestingly, PBC_MG99 has two regions where GC skew is negative (from the 11.5 kb to 12.9 kb marks and 27 to 28.5 kb marks) which coincide with genes being transcripted in the opposite direction.

A similar region to PBC_MG99’s largest one is found in phage PBC_MG88 between the 14 kb and 17 kb marks. For both phages, genes associated with related functions seemed clustered together: it is especially striking for virion structure genes, as tail genes are grouped together and separated from the capsid genes group by head–tail adaptor genes.

Gene annotation revealed the presence of the following lysogeny-related genes: a recombinase, LexA repressor, XRE transcriptional regulator, phage antirepressor, and immunity to superinfection protein. The latter is not present in phage PBC_MG88. Although the presence of the genes does not imply their expression, it is likely that these two phages are temperate. XRE and LexA repressors are probably linked to lytic cycle activation when the host strain is under xenobiotic stress or undergoing DNA damage. The recombinase, which is likely to be involved in lysogenic to lytic cycle transition, could be controlled by XRE and LexA repressors.

Figure 7 shows the BLAST comparison between the two phage genomes. Most regions shared high sequence identity (>90%), with a maximum of 98%, whereas some regions displayed no detectable similarity. The overall genome identity was 94.72%, supporting their classification as closely related but distinct phages. Similarity was highest in regions encoding structural proteins, while divergence was mainly observed in accessory gene regions. Although PBC_MG99 contains only one additional CDS compared to PBC_MG88 (72 vs. 71) as shown in Table 1, the two genomes differ qualitatively in accessory gene composition (Table S2). PBC_MG99 encodes auxiliary metabolic genes (AMGs) such as 1-hydroxy-2-methyl-2-(E)-butenyl 4-diphosphate synthase and 3-hydroxyacyl-CoA dehydrogenase, along with a translation-related gene (arginine–tRNA ligase) and regulatory/host-interaction proteins, whereas PBC_MG88 carries alternative AMGs (3-phosphoshikimate 1-carboxyvinyltransferase) and other regulatory or recombination-associated accessory genes, highlighting distinct sets of auxiliary functions despite their close overall similarity. Based on their predicted functions, several of these accessory genes likely act as AMGs, host-derived metabolic elements that can redirect or enhance host bacterial pathways to support phage replication without being strictly essential [101]. Phages frequently acquire AMGs from their hosts, and these genes are often involved in amino acid, carbohydrate, and energy metabolism. Phage-encoded AMGs have been shown to augment host metabolic pathways, including carbon, sulfur, and nutrient fluxes, effectively increasing metabolic activity during infection and demonstrating that metabolic genes beyond core viral functions are common and potentially functional in optimizing conditions for viral replication [102,103]. For example, the detection of *3-hydroxyacyl-CoA dehydrogenase* encoding gene in the PBC_MG99 genome is intriguing, as this enzyme catalyzes a key step in fatty acid β-oxidation pathway, converting L-3-hydroxyacyl-CoA into 3-ketoacyl-CoA while generating NADH [104]. In bacteria, β-oxidation contributes to energy production and carbon flux by supplying reducing equivalents and acetyl-CoA. When encoded by a phage, this gene may function as an AMG and could represent a moron-like element; while not strictly required for replication, it could enhance host energy production and metabolic fluxes, thereby improving phage biosynthesis during infection. By influencing β-oxidation, the phage could increase the availability of NADH and metabolic intermediates, supporting nucleotide synthesis, replication, and overall biosynthetic activity. Similarly, the presence of host-derived 1-hydroxy-2-methyl-2-(E)-butenyl-4-diphosphate synthase gene in PBC_MG99 is notable, as it catalyzes a key step in the non-mevalonate pathway of isoprenoid biosynthesis [105]. In bacteria, this pathway supports synthesis of isoprenoids required for membrane lipids, quinones, and other metabolites [106]. When encoded by a phage, this gene may act as an AMG and may help sustain host isoprenoid fluxes needed for phage proliferation.

Overall, the presence of host-derived auxiliary metabolic genes (AMGs) suggests a potential role in modulating host metabolism to support phage replication. However, their functional relevance remains speculative without transcriptional or biochemical validation. Further studies are needed to determine their roles during infection.

No transposons or insertion sequences were detected. Neither genome of the two phages contained virulence-associated genes or antibiotic resistance genes.

**Figure 8:**
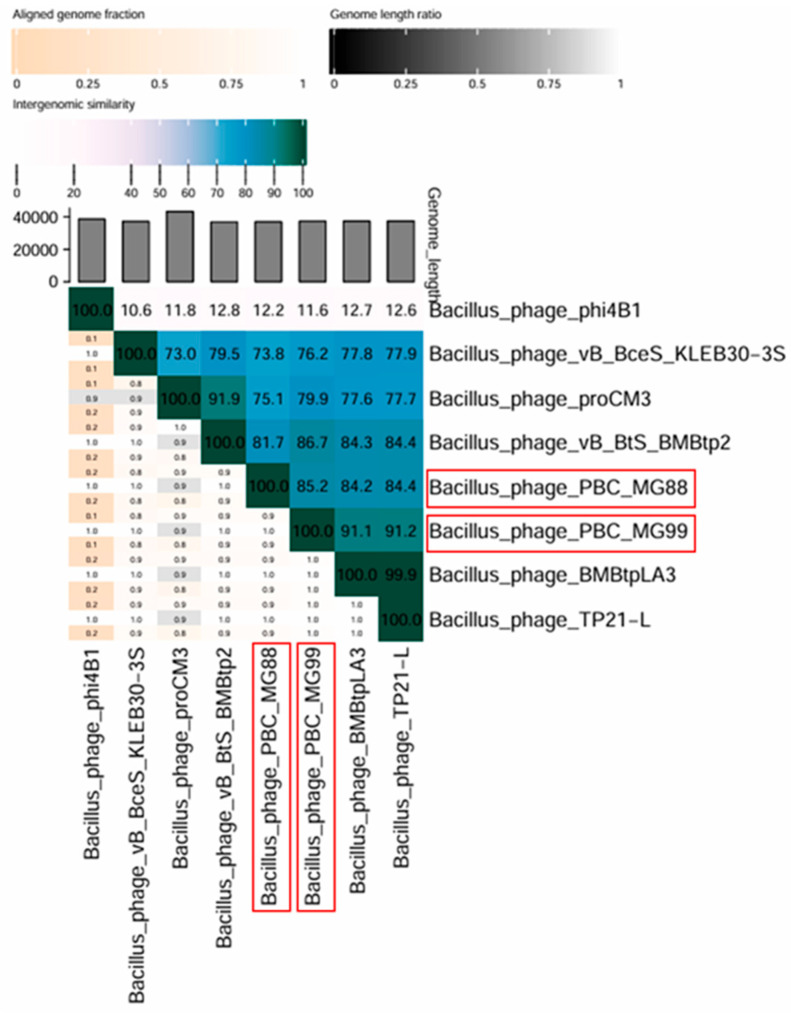
Heat map generated by VIRIDIC analysis showing the percentage of genome sequence similarity between phages. Phage names are indicated on the horizontal and vertical axes. Phages PBC_MG88 and PBC_MG99 are outlined by a red box.

Genome similarity was also evaluated using VIRIDIC (Figure 8). This analysis showed a maximum intergenomic similarity of 85.2% between the two phages. The lower value compared to BLAST results is expected, as BLAST only aligns highly similar regions, whereas VIRIDIC considers the entire genome, including regions with low or no similarity. In addition to intergenomic similarity, the heatmap generated by VIRIDIC v1.0 takes into account additional parameters, including the aligned fraction of each genome and the genome length ratio.

The genome similarity between PBC_MG88 and PBC_MG99 and the six most closely related phages in the NCBI database was below 95% (Figure 8). Among these, *Bacillus* phage TP21-L [107] showed the highest identity, with PBC_MG88 and PBC_MG99 sharing 84.4% and 91.2% genome sequence identity, respectively.

According to the International Committee on Taxonomy of Viruses (ICTV), the species demarcation threshold is 95% genome sequence identity, while the genus threshold is 70% [108]. Based on these criteria, PBC_MG88 and PBC_MG99 can be classified as novel species within the genus *Lwoffvirus*, displaying a siphovirus morphotype.

### 3.3. Effects of Phages on Biofilm Formation and Eradication

Based on the criteria of Stepanović et al. (2000) [97], both *B. cereus* strains 478 and 3990 were classified as strong biofilm producers at 24 h and 48 h. Total biofilm biomass, quantified by crystal violet staining (OD_595_), ranged from 0.75 ± 0.03 to 1.41 ± 0.02 and consistently exceeded fourfold the ODc (0.10 ± 0.01), thereby demonstrating a marked and time-dependent biofilm development. These findings validate biofilm establishment under the assay conditions, supporting the reliability of the subsequent phage inhibition and eradication experiments and ensuring that the observed reductions in OD accurately reflect true anti-biofilm activity.

**Figure 9:**
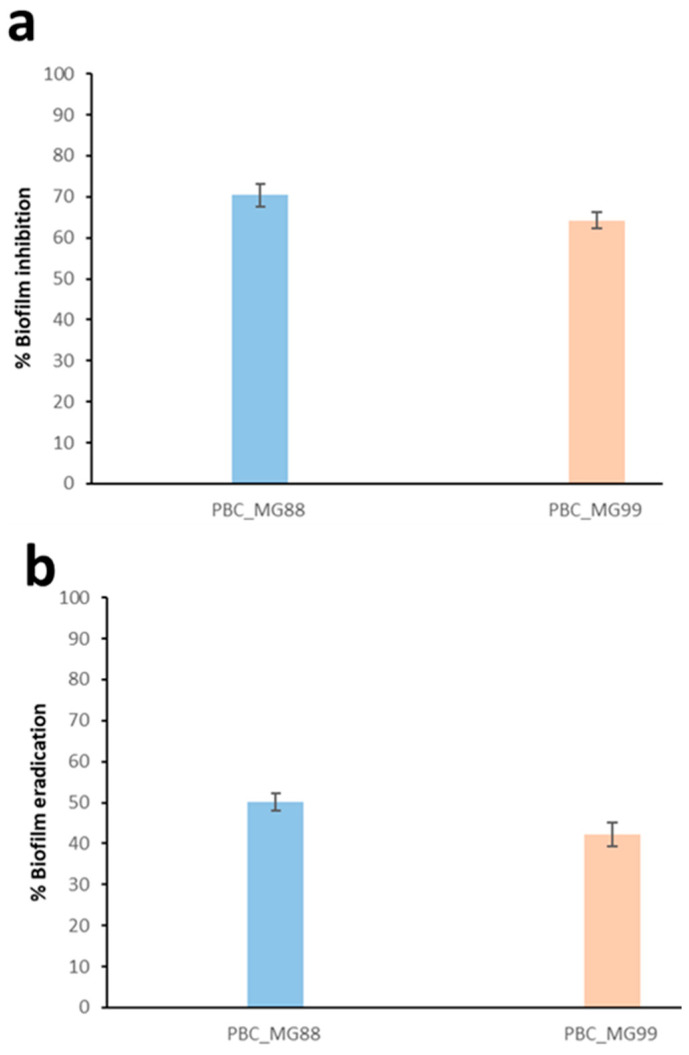
Effect of phages PBC_MG88 and PBC_MG99 on biofilms of *B. cereus* strains 3990 and 478, respectively. (**a**) Effect of phages PBC_MG88 and PBC_MG99 on inhibition of biofilms in 96-well microplates at 30 °C following 6 h incubation. (**b**) The removal effect of phage PBC_MG88 and PBC_MG99 on 48-h-old biofilm in 96-well microplates. Values represent the mean and standard deviation of three independent experiments.

To evaluate the ability of phages to prevent biofilm formation, each phage was added to the culture medium containing the host *B. cereus* strain prior to biofilm development. At a concentration of 10^7^ PFU/mL, corresponding to the optimal multiplicity of infection (MOI) of 0.1, both PBC_MG88 and PBC_MG99 significantly inhibited biofilm formation, resulting in reductions of approximately 70.3% and 64.2%, respectively (Figure 9a).

In addition, the ability of the phages to disrupt preformed biofilms was assessed. Treatment of 48-h-old biofilms with PBC_MG88 and PBC_MG99 led to reductions in biofilm biomass of 50.1% and 42.3%, respectively (Figure 9b).

## 4. Discussion

In this study, we isolated and characterized two *B. cereus* phages, PBC_MG88 and PBC_MG99. Transmission electron microscopy revealed that both phages belong to the class *Caudoviricetes* and exhibit siphovirus-like morphology (Figure 1c,f).

According to the ICTV classification criterion, the genomes of PBC_MG88 and PBC_MG99 shared less than 95% nucleotide identity with their six closest relatives in the NCBI database. The highest sequence identity was observed with *Bacillus* phage TP21-L [107], a member of the genus *Lwoffvirus*. Hence, the two phages might be a new species under the *Lwoffvirus* genus.

Genomically PBC_MG88 and PBC_MG99 share a common genome organization. Genes are grouped according to their function, in the same order, and genome size is identical within margin of error (37 026 bp for PBC_MG88 and 37 328 bp for PBC_MG99). This genome size is comparable to several previously reported *Bacillus* phages, including Deep-Purple (36,278 bp) [109], PBC1 (41.2 kb) [110], or vB_BceP_LY3 (28,124 bp) [35]. In contrast, some *Bacillus* phages carry considerably expanded genomes, such as DC1 (156,018 bp) and DC2 (155,908 bp) [30], Thurquoise (157,500 bp) [111], vB_BceM-HSE3 (124,002 bp) [112], ΦBc24 (160,311 bp) [113] and SWEP1 (162,461 bp) [114].

In line with observations in other bacteriophages, strong genome synteny and modular organization are widely recognized as hallmark features of phage genomes, and our two sequenced phages conform to this pattern [115,116,117]. In addition, phages infecting the same host species often draw from a shared genetic pool [63], which may account for the substantial similarity in gene content between PBC_MG88 and PBC_MG99.

Although PBC_MG88 and PBC_MG99 share significant genomic similarity, their biological characteristics differed, as revealed by one-step growth curve analysis used to determine essential phage infection parameters.

Phage latent period and burst size determines their potential application for use in biocontrol. A phage large burst size and short latent period will be beneficial in suppressing bacteria proliferation and disease outbreak [78]. PBC_MG88 exhibited a shorter latent period (≈20 min) and a moderate burst size (59 PFU/cell), whereas PBC_MG99 showed a slightly longer latent period (≈25 min) accompanied by a higher burst size (63 PFU/cell).

In comparison with other reported *Bacillus cereus* phages, including DC1 and DC2 [30]; vB_BceP-LY3 [35], ΦBc24 [113], and SWEP1 [114], the isolated phages displayed infection kinetics that are within the range reported for other *B. cereus* phages, supporting their potential applicability in biocontrol contexts.

The optimal MOI identified for PBC_MG88 and PBC_MG99 (0.1) differs from the MOI values reported for other *Bacillus cereus* phages, including vB_BceP-LY3 (0.0001) [35], ΦBc24 (0.01) [113], DC2 (1) and DC1 (10) [30], reflecting the variability in infection dynamics among phages infecting this species.

The ability of PBC_MG88 and PBC_MG99 to establish infection at relatively low phage-to-bacterium ratios may be relevant for practical applications, as effective propagation at low initial MOI has been reported to facilitate phage production and large-scale application while potentially reducing production costs [118].

Both phages showed rapid adsorption to host cells, with more than 90% of particles adsorbed within 15 min, indicating high adsorption efficiency, which is advantageous for their application in phage therapy.

Host range assays demonstrated that PBC_MG88 and PBC_MG99 were able to lyse multiple strains belonging to the *B. cereus* group, producing both clear and opaque plaques. The observation of clear and opaque plaques may reflect differences in phage–host interactions or lytic efficiency among strains [119]. Phage host specificity is generally determined by the presence of specific bacterial receptor structures [120]. In siphophages, receptor-binding proteins are typically associated with tail structures, including straight tail fibers or spike-like appendages located on the tail or baseplate [120,121].

For effective application, bacteriophages must remain stable under diverse environmental conditions. Temperature strongly influences phage adsorption, genome injection, replication, and latency [122], while pH affects stability and infectivity, with acidic conditions often leading to aggregation or inactivation [123,124]. In this study, PBC_MG88 and PBC_MG99 remained stable between 4 and 55 °C and across a pH range of 4–11. This stability is comparable to that of phages vB_BceP-LY3 (4–60 °C; pH 4–11) [35] and SWEP1 (<50 °C; pH 4–11) [114], broader than that reported for DC1 and DC2 (4–45 °C; pH 4–9) [30], and indicates higher thermal tolerance than ΦBc24 (≤50 °C; pH 2–12) [113]. Overall, these characteristics place PBC_MG88 and PBC_MG99 within the upper stability range described for *Bacillus cereus* phages. The environmental stability of PBC_MG88 and PBC_MG99 (4–55 °C and pH 4–11) not only matches but clearly exceeds the active growth limits of vegetative *B. cereus*, which grows between 8–50 °C (optimum 30–42 °C) and mainly within pH 5–7.5 [125]. Because phages cannot infect dormant spores, productive infection occurs only after spore germination and the re-establishment of vegetative metabolism [126]. Importantly, PBC_MG88 and PBC_MG99 remain stable under conditions that restrict *B. cereus* growth (e.g., pH 4 or low temperatures), allowing them to persist while spores are dormant. Once environmental conditions become host-permissive (≥pH 5 and temperatures within the growth range), vegetative cells emerge and are immediately susceptible to infection. The phages’ thermal and pH stability thus aligns with the physiological requirements for spore germination and bacterial proliferation, supporting their practical use as biocontrol agents in variable environments, including food systems [26].

After 24 h of phage incubation on a lawn of the host *B. cereus* strain, halo formation was observed around the phage plaques. This halo phenomenon has been reported only once previously for *B. cereus* phages, where phages DC1 and DC2 produced plaques surrounded by a fuzzy halo [30].

The formation of halos around phage plaques suggests the possible presence of depolymerase activity, as such enzymes are known to degrade polymers on the bacterial surface [109,127,128]. However, no depolymerase genes were annotated in the genomes of PBC_MG88 and PBC_MG99, indicating that these phages may encode yet-unidentified depolymerases or enzymes with similar functions.

Phages possessing depolymerase or depolymerase-like activity can gain access to host cell receptors by degrading capsular polysaccharides and extracellular polymeric substances. This activity is associated with enhanced antibiofilm efficacy, as polymer degradation may facilitate phage penetration and access to bacterial cells located in different layers of the biofilm [129,130,131,132]. Such properties are particularly relevant in food-processing environments, where biofilms contribute significantly to persistent microbiological contamination.

In this study, phages PBC_MG88 and PBC_MG99 demonstrated strong antibiofilm activity, effectively inhibiting biofilm formation and degrading established biofilms. These findings suggest that the phages acted as efficient biofilm scavengers, likely due to depolymerase-mediated matrix degradation.

Previous studies have reported similar antibiofilm effects of phages against foodborne pathogens, including reductions in *Escherichia coli* [133,134], *Salmonella* [135,136,137,138,139], *Enterococcus faecalis* [140], *Listeria monocytogenes* [141,142], *Pseudomonas fluorescens* [143], *Staphylococcus aureus* [144], and *Streptococcus mutans* [145] biofilms. However, bacteriophage-based strategies for controlling *B. cereus* biofilms remain poorly explored, with only two studies reported to date [30,31], revealing a significant knowledge gap in this area. Therefore, this study provides valuable evidence supporting the potential of phages as effective biocontrol tools for the prevention and control of *B. cereus* biofilms in food-related settings and supports their further development as alternative antibiofilm approaches. Importantly, in the case of emetic *B. cereus*, the main health risk is the heat-stable preformed cereulide that persists in food after processing, rather than from active infection. Biofilms play a central role in Bacillus cereus contamination and the spread of cereulide in food environments. This stable surface-associated lifestyle not only allows bacteria to persist, but also creates conditions that promote toxin synthesis [146]. In this context, biofilms are intrinsically linked to cereulide production, with regulators such as CodY and AbrB controlling both toxin synthesis and biofilm growth. When these regulators are downregulated, cereulide production may increase alongside biofilm development [147,148]. Moreover, cereulide may function in cell-to-cell communication via quorum sensing, coordinating biofilm formation, maturation, and disassembly in response to cell density [149,150]. Together, these features allow biofilms to maintain bacteria and toxins on surfaces, increasing the risk of contamination and the transfer of cereulide to food.

Additionally, *B. cereus* cells within biofilms release metabolites and toxins directly into the biofilm matrix, where cereulide tends to remain bound rather than dispersing into the environment [148,151]. As a result, residual biofilm and associated cereulide can persist on surfaces even after cleaning, creating the potential for direct contact between food and the surviving toxin-containing biofilm. Preventive measures aimed at controlling the growth of *B. cereus* and limiting cereulide production are critical to mitigating the risk of emetic food poisoning [152]. This requires strict temperature management during preparation and storage, effective prevention and removal of biofilms from food-contact surfaces, and minimization of cross-contamination across the entire food chain [153]. In this context, phage application represents a promising complementary strategy to strengthen current control measures. By targeting vegetative cells of emetic *B. cereus*, including those embedded within biofilms where cereulide may accumulate, phages can significantly reduce bacterial loads and disrupt biofilm-associated reservoirs. Although they do not inactivate pre-formed cereulide, their capacity to prevent further bacterial growth effectively limits additional toxin production prior to consumption. Therefore, phages such as PBC_MG88 and PBC_MG99 should be regarded as preventive biocontrol agents that enhance food safety interventions and reinforce risk mitigation throughout the food chain. At the same time, phages pose several challenges in their application as antimicrobial agents in the food industry [154]. These include the insurance of their stability and effectiveness, preventing transduction, minimizing the development of phage-resistant bacteria, and avoiding the horizontal gene transfer of undesirable traits of antibiotic resistance or virulence. These limitations can be mitigated through careful genomic screening to mitigate these risks and ensure their safe application in the food industry.

On the basis of genome sequencing, PBC_MG88 and PBC_MG99 were classified as temperate phages due to the presence of genes associated with both lysogenic and lytic cycles. Genome analysis revealed no detectable genes encoding antibiotic resistance, toxins, or known virulence factors.

Temperate phages are often considered less suitable as antibacterial agents because of their potential to mediate horizontal gene transfer, including the dissemination of bacterial virulence or antibiotic resistance genes [66,72].

Nevertheless, multiple studies have described strategies to harness temperate phages while minimizing associated risks [70,71]. For example, temperate phages can be genetically modified to enforce obligate lytic activity or used in combination with antibiotics that suppress lysogen formation, enhancing bacterial clearance, disrupting biofilms, and even functionally re-sensitizing resistant strains by reducing antibiotic minimum inhibitory concentrations [28].

In addition, the introduction of specific genetic modules into temperate phages has been shown to induce programmed bacterial cell death, potentially reducing the reliance on conventional antibiotics and their associated toxic effects [72].

Beyond these approaches, temperate phages have been reported to display enhanced activity against intracellular bacteria compared with strictly virulent phages, which often fail to access intracellular targets [73]. As a result, temperate phages have been applied therapeutically either in their native form or following genetic modification to treat a variety of bacterial infections [155]. Alternatively, individual genes derived from temperate phages that confer bactericidal activity have been isolated and repurposed for antibacterial applications [156].

Overall, this study underscores the importance of genome sequencing in evaluating phage candidates for antibacterial applications. Although PBC_MG88 and PBC_MG99 displayed several lytic-like features and potential for *B. cereus* biofilm control, the presence of lysogeny-associated genes indicates a temperate lifestyle, suggesting that additional steps may be required before therapeutic use.

## 5. Conclusions

In this study, the biological characteristics and genomic features of two *B. cereus* phages, PBC_MG88 and PBC_MG99, were investigated. Both phages displayed strong lytic ability against *B. cereus* strains and good stability under the tested conditions. In addition, they showed the ability to reduce *B. cereus* biofilms, indicating potential relevance for biocontrol applications in the food industry. Genome analysis revealed the absence of genes associated with antibiotic resistance or known virulence factors. However, the presence of lysogeny-related genes suggests a temperate lifestyle, which may raise safety considerations for direct application.

Further investigations are warranted to better characterize lysogeny-associated functions, assess genomic stability, and evaluate potential safety considerations relevant to the use of temperate phages, including regulatory and human health aspects.

## Figures and Tables

**Table 1:** Assembly and annotation statistics for the sequenced phages.

Phage	Assembly Size (bp)	Coverage	Number of CDS
PBC_MG88	37 026	X 6869	71
PBC_MG99	37 328	X 3808	72

## Data Availability

The original contributions presented in this study are included in the article. Further inquiries can be directed to the corresponding author. The whole-genome sequences were deposited in the GenBank database under the accession numbers PQ618007 (PBC_MG88) and PQ634339 (PBC_MG99).

## Supplementary Materials

The following supporting information can be downloaded at: https://www.mdpi.com/article/10.3390/v18030306/s1, Table S1: Host range analysis of PBC_MG88 and PBC_MG99. Host range was determined by a spot test assay against 172 *Bacillus cereus* group strains. Three lysis patterns were observed and are shown in this matrix: lysis with clear plaque (yellow), lysis with opaque plaque (green), and no lysis (gray). Table S2: Shared and unique gene annotations of PBC_MG88 and PBC_MG99. Gene presence was determined via genome annotation. Genes specific to each phage (unique) are highlighted in gray, while genes shared by both phages are unshaded.
