# Supplementary material for: Isolation, Characterization and Genomic Analysis of PBC_MG88 and PBC_MG99 Bacteriophages and Their Antibiofilm Activity Against the Bacillus cereus Groups
Source: Viruses. 2026 Feb 28;18(3):306. doi: 10.3390/v18030306 (PMC13030324; doi:10.3390/v18030306)
Supplement: Supplementary file 1 [file viruses-18-00306-s001.zip › viruses-4153345-supplementary.pdf]

Table S1: Host range analysis of PBC_MG88 and PBC_MG99. Host range was determined by a spot test assay against 172 *Bacillus cereus* group strains. Three lysis patterns were observed and are shown in this matrix: lysis with clear plaque (yellow), lysis with opaque plaque (green), and no lysis (gray).

No.	Strain	Sensitivity		No.	Strain	Sensitivity		No.	Strain	Sensitivity	
		PBC_MG88	PBC_MG99			PBC_MG88	PBC_MG99			PBC_MG88	PBC_MG99
1	C14			61	P4			121	5674		
2	C23			62	396			122	P1 19j		
3	156			63	3828 pc			123	P1 8j		
4	228			64	3995			124	p7		
5	2878			65	3997			125	168		
6	3298			66	4005			126	2885		
7	3299			67	4071			127	4006		
8	3607			68	4086			128	5664		
9	3828 gc			69	2884			129	5666		
10	3938 gc			70	2889			130	C21		
11	3940 gc			71	2893			131	4947 pc		
12	3942 gc			72	3042			132	C22		
13	3949			73	3047			133	C25 gc		
14	3996			74	4000			134	P2 13j		
15	4309 8j			75	4855			135	2788		
16	4309 13j			76	C12			136	2883		
17	4727			77	C13			137	3611		
18	5456			78	C24			138	4007		
19	5659			79	2845			139	4044		
20	2964			80	4950			140	4160		
21	2976			81	5380 8j			141	4167		
22	3199			82	5655			142	SF2 gc		
23	4068 13j			83	5662 7j			143	2873		
24	4068 7j			84	C28			144	C17		
25	3296			85	Cous GC			145	C20		
26	4313			86	E2			146	C26		
27	5390			87	E10			147	E7		
28	5665			88	4165			148	E19		
29	C18 gc			89	4478			149	SF2 pc		
30	499			90	4785			150	2875		
31	4664			91	5603			151	2876		
32	5657			92	5662 13j			152	2881		
33	C25 pc			93	E1			153	2900		
34	2975			94	E16			154	2957 13j		
35	4927 pc			95	P3 13j			155	2981		
36	4927 gc			96	P3 7j			156	2982		
37	C10			97	P6			157	3041		
38	2770			98	SF1 gc			158	3051		
39	2892			99	207			159	4084		
40	3988 pc			100	211			160	4168		
41	2772			101	227			161	4171		
42	3297			102	500			162	4220		
43	5002			103	4663			163	4253 pc		
44	171			104	P5			164	4314		
45	225			105	SF1 pc			165	4315		
46	3050			106	SR gc			166	4316		
47	3988 gc			107	159			167	4477		
48	5669			108	160			168	4593		
49	2789			109	678			169	4788		
50	4931			110	2787			170	4830		
51	3942 pc			111	2874			171	4920 pc		
52	4163			112	2956			172	5600		
53	C18 pc			113	3197						
54	P2 7j			114	3201						
55	842			115	4085						
56	3940 pc			116	4253 gc						
57	5728			117	4920 gc						
58	3938 pc			118	C27						
59	4087			119	4851						
60	4947 gc			120	5380 13j						

Table S2: Shared and unique gene annotations of PBC_MG88 and PBC_MG99. Gene presence was determined via genome annotation. Genes specific to each phage (unique) are highlighted in gray, while genes shared by both phages are unshaded.

Genes annotation	PBC_MG99	PBC_MG88
N-acetylmuramoyl-L-alanine amidase	+	
1-hydroxy-2-methyl-2-(E)-butenyl 4-diphosphate synthase	+	
3-hydroxyacyl-CoA dehydrogenase	+	
3-phosphoshikimate 1-carboxyvinyltransferase		+
Arginine--tRNA ligase	+	
Bacteriocin-like protein	+	+
Baseplate J-like protein	+	+
Baseplate subunit	+	+
DEAD/DEAH box helicase		+
DNAB-Like Replicative Helicase	+	+
Head completion protein	+	+
Head morphogenesis protein	+	+
Head-tail adaptor	+	+
Holin	+	
Host-nuclease inhibitor protein Gam	+	+
Imm immunity to superinfection membrane protein	+	
Late sigma factor		+
LexA family transcriptionnal regulator		+
Major capsid protein	+	+
Minor structural protein	+	+
N-acetylmuramoyl-L-alanine amidase		+
NADH dehydrogenase I subunit C/D	+	
Nuclease	+	+
Phage antirepressor	+	+
Phage repressor		+
Phage repressor protein	+	
Portail protein	+	+
Recombinase	+	+
Recombination protein		+
RNA polymerase sigma-70 factor	+	
RNA polymerase subunit beta	+	
RNA-DNA and DNA-DNA helicase	+	
Scaffold protein	+	
Tail protein	+	+
Tail tape measure protein		+
Tape measure protein	+	
Terminase large subunit	+	+
TM2 Domain containing protein	+	+
XRE family transcriptionnal regulator	+	+
Hypthotetical protein	35	39

"+" indicates gene presence.
